# Dynamic Behavior of Carbon Nanotube-Reinforced Polymer Composite Ring-like Structures: Unraveling the Effects of Agglomeration, Porosity, and Elastic Coupling

**DOI:** 10.3390/polym17050696

**Published:** 2025-03-05

**Authors:** Hossein Mottaghi T., Moein A. Ghandehari, Amir R. Masoodi

**Affiliations:** Department of Civil Engineering, Ferdowsi University of Mashhad, Mashhad 9177948974, Iran; hossein.mottaghi@mail.um.ac.ir (H.M.T.); ma.ghandehari@mail.um.ac.ir (M.A.G.)

**Keywords:** coupled beam ring, vibration, carbon nanotube (CNT), agglomeration, generalized differential quadrature method (GDQM), elastic springs

## Abstract

This research examines the free vibration characteristics of composite ring-like structures enhanced with carbon nanotubes (CNTs), taking into account the effects of CNT agglomeration. The structural framework comprises two concentric composite rings linked by elastic springs, creating a coupled beam ring (CBR) system. The first-order shear deformation theory (FSDT) is applied to account for transverse shear deformation, while Hamilton’s principle is employed to formulate the governing equations of motion. The effective mechanical properties of the composite material are assessed with regard to CNT agglomeration, which has a significant impact on the elastic modulus and the overall dynamic behavior of the structure. The numerical analysis explores the influence of porosity distribution, boundary conditions (BCs), and the stiffness of the springs on the natural vibration frequencies (NVFs). The results demonstrate that an increase in CNT agglomeration leads to a reduction in the stiffness of the composite, consequently decreasing the NVFs. Furthermore, asymmetric porosity distributions result in nonlinear fluctuations in NVFs due to irregularities in mass and stiffness, whereas uniform porosity distributions display a nearly linear relationship. This study also emphasizes the importance of boundary conditions and elastic coupling in influencing the vibrational response of CBR systems. These findings offer significant insights for the design and optimization of advanced composite ring structures applicable in aerospace, nanotechnology, and high-performance engineering systems.

## 1. Introduction

Rings are fundamental structural elements extensively employed in various engineering disciplines due to their unique behavior and geometric properties. These components are integral in aerospace applications, such as rocket casings and fuselage sections, and in automotive systems like piston rings and flywheels, where their high strength-to-weight ratio and rotational symmetry are leveraged for efficiency and performance. Additionally, rings serve a critical role in modeling complex mechanisms, including planetary gear trains and driveline dampers, owing to their ability to distribute loads uniformly and simplify analytical and computational models. While their advantages include exceptional circumferential stress resistance and adaptability to diverse applications, challenges arise in addressing stress concentrations under localized or non-uniform loads, which may lead to fatigue or failure. Furthermore, manufacturing rings, especially in large sizes or composite materials, can pose technical and economic difficulties. Despite these challenges, rings remain indispensable in engineering design due to their versatility and effectiveness in demanding applications. Abedinilaksar and Yang [1] explore the free in-plane vibration of thin-walled rings supported by elastic elements. The model employs Euler–Bernoulli theory under the inextensibility assumption, with linear springs simulating real-world bolted supports. A closed-form solution is derived for NVFs and mode shapes. Notably, tangential stiffness has a greater impact on vibration behavior compared to radial and torsional stiffness. Wu and Parker [2] analyze the free vibration eigen solutions of thin rings on elastic foundations with varying stiffness. Perturbation and Galerkin methods are utilized to derive closed-form expressions for NVFs and vibration modes. Xu et al. [3] examine vibration characteristics of FGM rings modeled under plane stress and strain assumptions. A theoretical framework is developed using first-order shear deformation theory and von Kármán relations. Wang et al. [4] present a theoretical analysis of ring vibrations through a wave-based approach. Propagation and reflection matrices are used to derive NVFs, aligning with classical and FEM results.

Multilayered composite materials (MCMs) are engineered materials composed of multiple layers, or laminae, of distinct materials bonded together to create a composite structure with tailored mechanical, thermal, or chemical properties. Each lamina typically consists of nanofiller or nanofiber embedded in a polymer matrix, which provides strength, stiffness, and durability. By arranging these layers in specific orientations, laminated composites achieve enhanced performance characteristics that exceed those of their individual components. These materials are widely used in aerospace, automotive, marine, and civil engineering applications due to their high load-bearing efficiency, excellent fatigue resistance, and customizable properties [5,6]. Various studies utilize these structures for different structural elements. Garg et al. [7] investigate vibration behavior of helicoidal multilayered composite plates using isogeometric analysis-based refined theory. Karamanli et al. [8] analyze mechanical behaviors of bio-inspired helicoidal multilayered composite beams using the Ritz method. Ghandehari et al. [9] explore the vibration behavior of multilayer conical shells. Jiang et al. [10] investigate the dynamic behavior and energy distribution in acoustic black hole multilayered beams with elastic boundary conditions. Deng et al. [11] examine the flexural performance and design methodology of multilayered H-beams in modular steel construction (MSC). Zhang et al. [12] investigate the vibration characteristics of a laminated composite double-plate system using the FSDT and classical delamination theory. Sahib and Kovács [13] model composite sandwich structures using an artificial neural network. The study employs Monte Carlo sampling and an FEM to generate training data, considering various design variables such as face sheet materials, layer numbers, core types, and load magnitudes.

Vibration is a phenomenon that influences the performance and stability of structures. In engineering, understanding and controlling vibration is essential for optimizing efficiency, comfort, and safety. By analyzing and predicting vibrational responses, engineers can design systems that either exploit or mitigate vibrations, ensuring optimal behavior under dynamic loading conditions. In free vibration, the system’s motion is solely governed by its intrinsic properties, such as mass, stiffness, and damping. Therefore, exploring the vibration behavior of structures is vital step in designing. Alkhoury et al. [14] integrate finite element (FE) structural analysis with observer-based active control to evaluate the real-time performance of an Active Tuned Mass Damper in reducing fore–aft vibrations in monopile-supported offshore wind turbines. Avey et al. [15] explore the nonlinear vibration of moderately thick multilayer shell-type structural elements with double curvature, featuring CNT patterned layers, using generalized FSDT. Mottaghi et al. [16] investigate the vibrational response of curved composite beams enhanced with CNTs, utilizing a multiscale FEM to examine bending, shear deformations, and NVFs. Liu et al. [17] investigate the vibration characteristics of the propulsion shaft-combined shell system. Thang et al. [18] explore the free vibration characteristics of honeycomb sandwich cylindrical shells reinforced with graphene nanoplatelets (GNPs), using the refined trigonometric shear deformation theory to capture both bending and shear deformations.

In recent years, various numerical methods have been presented to calculate the NVFs of structural elements. For example, FEM [19], Rayleigh–Ritz [20], the differential quadrature method (DQM) [21], integral quadrature method (IQM) [22], and generalized differential quadrature method (GDQ) [23]. This investigation utilized the GDQ method for calculation of NVFs of CBRs. This method has gained significant attention due to its advantages in solving complex structural problems. One of the key pros of the GDQ method is its ability to provide high accuracy with relatively low computational cost, especially for problems with complex boundary conditions and geometries. GDQ is highly efficient in discretizing continuous functions, making it ideal for analyzing vibration, dynamic, and stability problems in structural elements like composite beams. One drawback is the difficulty in handling nonlinearity, especially when material properties or BCs change significantly during the analysis.

This study aims to investigate the vibrational behavior of CBRs. The CBR is a complex structural model consisting of two rings connected by a layer of elastic springs, constructed from laminated composite materials. The novelty of this work lies in the development of a novel model for CBRs, providing a deeper understanding of their vibrational behavior. In the case of composite materials, the effect of agglomeration on the NVFs is examined. Various numerical examples are presented to enhance the understanding of the NVFs and dynamic behavior of this system

## 2. Ring Mathematical Modeling

**Figure 1:**
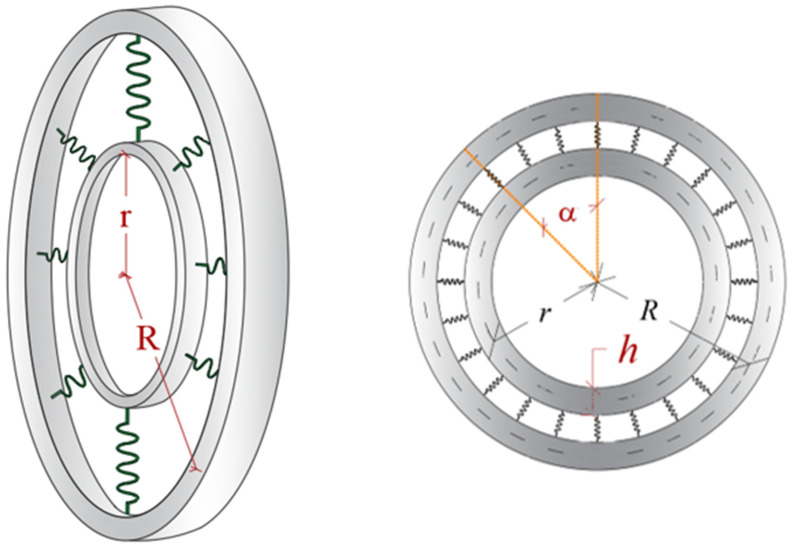
The definition of the geometry of the CBR.

CBRs combine two rings that are connected to each other with a layer of elastic springs. For mathematical modeling of this system, the beam theory is utilized. The geometrical properties of this system are defined with the following: R radius of outer beam, r radius of inner beam, L the length of outer ring, l the length of inner ring, α the central angle, and h and b are the height and width of the ring’s section, respectively. Figure 1 presents a schematic of this system.

**Figure 2:**
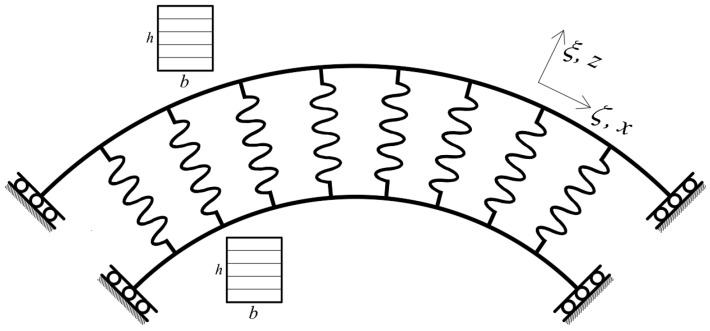
The definition of the geometry of the QCBR.

In this simulation, a quarter of a CBR (QCBR) is modeled for investigation of NVFs. The model utilized in the mathematical modeling is presented in Figure 2. The geometric configuration of this scheme of model is *α* = π/2. It is worth mentioning that the BCs of the QCBR, in this way of modeling, are shear-diaphragm.

In modeling, to associate displacements to strains, FSDT is employed as follows:

(1) $$\left\{\begin{array}{l} Z(x,\;z,\;t)=\zeta (x,t)+z\;\Phi (x,\;t)\\ \Xi (x,\;z,\;t)=\xi (x,t) \end{array}\right.\;$$

Here, the displacement in the longitude direction (*x*) are represented with *Z* and in and the thickness (*z*) direction shown with *Ξ*. The displacement of the reference layer in the longitude (*x*) and thickness (*z*) directions are shown with *ζ* and *ξ*, respectively. *Φ* is an indicator for the reference layer displacement. To associate the strain of the ring and reference layer with the curvature of ring, a cylindrical panel scheme (CPS) and FSDT are utilized.

(2) $$\left\{\begin{array}{c} \varepsilon_{x}\\ \gamma_{xz} \end{array}\right\}=\left[\begin{array}{ccc} \lambda_{1} & \lambda_{2} & 0\\ 0 & 0 & \lambda_{1} \end{array}\right]\;\left\{\begin{array}{c} \varepsilon_{x}^{M}\\ \phi \\ \gamma_{xz}^{M} \end{array}\right\}\;$$

Here, $\varepsilon_{x}$ and $\gamma_{xz}$ are the normal and shear strains of the ring. The reference normal and shear strains are represented with $\varepsilon_{x}^{M}$, and $\gamma_{xz}^{M}$, respectively. *ϕ* is a factor associated to the curvature of the ring. Moreover,

(3) $$\lambda_{1}=\frac{1}{1+\beta },\;\lambda_{2}=\frac{z}{1+\beta }\;$$

where *b* is the ratio of *z* to the radius of the ring. To associate reference strain to the displacement of the ring the following relation is considered.

(4) $$\left\{\begin{array}{c} \varepsilon_{x}^{M}\\ \phi \\ \gamma_{xz}^{M} \end{array}\right\}=\left[\begin{array}{ccc} \frac{\partial }{\partial x} & \frac{1}{R} & 0\\ -\frac{1}{R} & \frac{\partial }{\partial x} & 1\\ 0 & 0 & \frac{\partial }{\partial x} \end{array}\right]\;\left\{\begin{array}{c} \zeta \\ \Phi \\ \xi \end{array}\right\}$$

To establish a relation between stresses and strains of a ring, Hook’s law is utilized.

(5) $$\left\{\begin{array}{c} \sigma_{x}\\ \tau_{xz} \end{array}\right\}=\left[\begin{array}{cc} \beta_{1} & 0\\ 0 & \beta_{2} \end{array}\right]\;\left\{\begin{array}{c} \varepsilon_{x}\\ \gamma_{xz} \end{array}\right\}\;$$

In this study, an MCM considered for rings. It is assumed that layers are orthotropic, and the *β*_1_ and *β*_2_ are calculated from the following equations:

(6) $$\begin{array}{l} \frac{1}{\beta_{1}}=\frac{\cos{\left(\varphi \right)}^{4}}{E_{L}^{11}}+\left[\frac{1}{G_{L}^{12}}-2\;\frac{\nu_{L}^{12}}{E_{L}^{11}}\right]\;\cos{\left(\varphi \right)}^{2}\;\sin{\left(\varphi \right)}^{2}+\frac{\sin{\left(\varphi \right)}^{4}}{E_{L}^{22}}\\ \frac{1}{\beta_{2}}=\frac{\cos{\left(\varphi \right)}^{2}}{G_{L}^{13}}+\frac{\sin{\left(\varphi \right)}^{2}}{G_{L}^{23}} \end{array}\;$$

Here, $E_{L}^{11}$, $E_{L}^{22}$, and $G_{L}$ are axial Young’s modulus, transverse Young’s modulus, and shear modulus, respectively. By a numerical integration along the thickness (*z*) direction of the ring, the forces and moments can be determined.

(7) $$N=b\int_{-0.5h}^{0.5h}\sigma_{x}\;dz,\;V=b\int_{-0.5h}^{0.5h}\tau_{xz}\;dz,\;M=b\int_{-0.5h}^{0.5h}z\sigma_{x}\;dz$$

Here, *N*, *V*, and *M* are normal force, shear force, and moment, respectively. By substituting Equation (17) into Equations (15) and (12), a relation between forces and reference strains can be established.

(8) $$\begin{array}{l} N=\;\phi \;B_{11}+\varepsilon_{x}^{M}\;A_{11}\\ V=\;\gamma_{xz}^{M}\;A_{55}\\ M=\;\phi \;D_{11}+\;\varepsilon_{x}^{M}\;B_{11} \end{array}\;$$

where

(9) $$A_{11}=\;R\;b\int_{-0.5h}^{0.5h}\left(\frac{E_{MCM}}{R+z}\right)\;dz,\;B_{11}=\;R\;b\int_{-0.5h}^{0.5h}\left(\frac{E_{MCM}\;}{R+z}\right)z\;dz,\;D_{11}=\;R\;b\int_{-0.5}^{0.5}\left(\frac{E_{HCS}\;}{R+z}\right)z^{2}\;dz,\;A_{55}=\;\kappa \;R\;b\int_{-0.5h}^{0.5h}\left(\frac{1}{R+z}\right)\;\left(\frac{E_{L}}{2\;(1+\nu_{L})}\right)\;dz\;$$

Here, κ is the shear correction factor, which is equal to 0.833. After determining the forces and strains in rings, it is time to establish the motion equation of the ring. For this purpose, Hamilton’s principal is utilized. The action integral is expressed as the time integral of the Lagrangian, which is the difference between the kinetic energy and the strain and potential energy of the system.

(10) $$\delta \int_{t_{1}}^{t_{2}}\left\{K-\left(Ρ+S\right)\right\}\;\mathrm{dt}=0$$

Here, δK, δΡ, and δS are virtual kinematic energy, virtual potential energy, and virtual strain energy, respectively. In the case of free vibration, the virtual potential energy is zero because there is no external loading. The following relation can be utilized to achieve virtual potential energy:

(11) $$\delta K=R\;b\;\int_{0}^{L}\int_{-0.5h}^{0.5h}\rho \left[{\left(\dot{\zeta }\right)}^{2}+{\left(\dot{\xi }\right)}^{2}\right]\;\left(\frac{1}{R+z}\right)\;dz\;dx$$

Here, (*) denotes the first-order derivation. For determining the virtual strain energy,

(12) $$\delta S=b\;\int_{0}^{L}\int_{-0.5h}^{0.5h}\left[\sigma_{x}\;\varepsilon_{x}+\tau_{xz}\;\gamma_{xz}\right]\;dz\;dx$$

**Figure 3:**
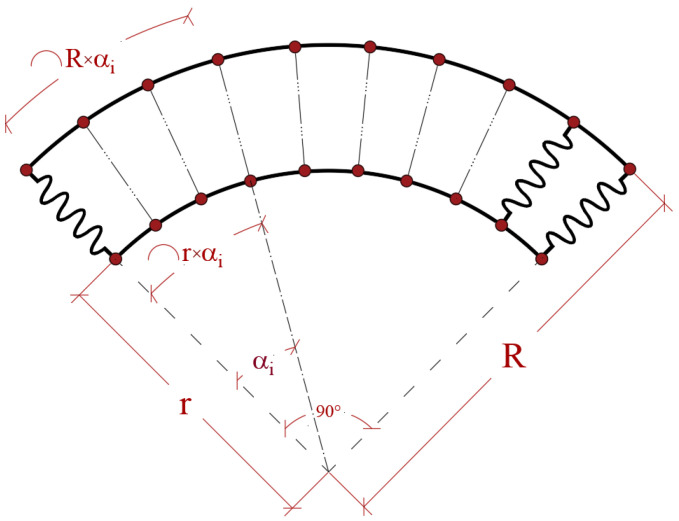
The definition of the geometry of the QCBR system.

By substituting Equations (21) and (22) in Equation (20), the motion equations of the rings are obtained. In this modeling, two different rings are connected to each other by a layer of elastic spring. To model this system, the springs should be perpendicular to the rings at each point. For this purpose, the grid point positions of the rings should correspond. Figure 3 presents the positions of rings and springs.

To ensure the springs are perpendicular to the ring surface, the following relation is developed for the grid points of the ring:

(13) $$\alpha_{i}=\frac{x_{i}^{in}}{r}$$

(14) $$x_{i}^{out}=\alpha_{i}R$$

Here, *a_i_* is the central angle of ring for each grid point of the inner ring ($x_{i}^{in}$). $x_{i}^{out}$ is a parameter showing the grid distribution of the outer ring. By developing motion equation for both rings and applying Equations (23) and (24), the motion equation of a CBR is obtained. By utilizing the Green–Gause theory and integration to motion equations, the GEMs of a CBR can obtained.

(15) $$\frac{1}{r}\left[V^{in}+r\;\frac{\partial N^{in}}{\partial x}\right]=I_{0}^{in}\;\frac{\partial^{2}\zeta }{\partial t^{2}}+I_{1}^{in}\;\frac{\partial^{2}\Phi }{\partial t^{2}}$$

(16) $$\frac{1}{r}\left[N^{in}+r\;\frac{\partial V^{in}}{\partial x}\right]+K_{mid}\;\left(\xi^{in}-\xi^{out}\right)=I_{0}^{in}\;\frac{\partial^{2}\xi }{\partial t^{2}}$$

(17) $$\frac{\partial M^{in}}{\partial x}-V^{in}=I_{1}^{in}\;\frac{\partial^{2}\zeta }{\partial t^{2}}+I_{2}^{in}\;\frac{\partial^{2}\Phi }{\partial t^{2}}$$

(18) $$\frac{1}{R}\left[V^{out}+R\;\frac{\partial N^{out}}{\partial x}\right]=I_{0}^{out}\;\frac{\partial^{2}\zeta }{\partial t^{2}}+I_{1}^{out}\;\frac{\partial^{2}\Phi }{\partial t^{2}}$$

(19) $$\frac{1}{R}\left[N^{out}+R\;\frac{\partial V^{out}}{\partial x}\right]+K_{mid}\;\left(\xi^{out}-\xi^{in}\right)=I_{0}^{out}\;\frac{\partial^{2}\xi }{\partial t^{2}}$$

(20) $$\frac{\partial M^{out}}{\partial x}-V^{out}=I_{1}^{out}\;\frac{\partial^{2}\zeta }{\partial t^{2}}+I_{2}^{out}\;\frac{\partial^{2}\Phi }{\partial t^{2}}$$

where

(21) $$I_{0}^{in}=\frac{b}{r}\;\left(\int_{-0.5h}^{0.5h}\rho \;z\;dz+r\;\;\int_{-0.5h}^{0.5h}\rho \;dz\right),\;I_{0}^{out}=\frac{b}{R}\;\left(\int_{-0.5h}^{0.5h}\rho \;z\;dz+R\;\;\int_{-0.5h}^{0.5h}\rho \;dz\right),\;I_{1}^{in}=\frac{b}{r}\;\left(\int_{-0.5h}^{0.5h}\rho \;z^{2}\;dz+r\;\;\int_{-0.5h}^{0.5h}\rho \;z\;dz\right),\;I_{1}^{out}=\frac{b}{R}\;\left(\int_{-0.5h}^{0.5h}\rho \;z^{2}\;dz+R\;\;\int_{-0.5h}^{0.5h}\rho \;z\;dz\right),\;I_{2}^{in}=\frac{b}{r}\;\left(\int_{-0.5h}^{0.5h}\rho \;z^{3}\;dz+r\;\;\int_{-0.5h}^{0.5h}\rho \;z^{2}\;dz\right),\;I_{2}^{out}=\frac{b}{R}\;\left(\int_{-0.5h}^{0.5h}\rho \;z^{3}\;dz+R\;\;\int_{-0.5h}^{0.5h}\rho \;z^{2}\;dz\right)\;$$

The presented implementation is employed to determine the angular frequencies (*ω*) of the CBR:

(22) $$\left\{\begin{array}{c} \zeta^{in}(x,z,t)\\ \Phi^{in}(x,z,t)\\ \xi^{in}(x,z,t)\\ \zeta^{out}(x,z,t)\\ \Phi^{out}(x,z,t)\\ \xi^{out}(x,z,t) \end{array}\right\}=e^{i\omega t}\;\left\{\begin{array}{c} F_{1}^{in}(x)\\ F_{2}^{in}(x)\\ F_{3}^{in}(x)\\ F_{1}^{out}(x)\\ F_{2}^{out}(x)\\ F_{3}^{out}(x) \end{array}\right\}\;$$

By considering Equations (21) to (25) and utilizing the Green–Gauss theory and integration, the GEMs are obtained as follows:

(23) $$\begin{array}{c} \frac{A_{11}^{in}}{r}\left(\frac{dF_{3}^{in}(x)}{dx}+r\;\;\frac{d^{2}F_{1}^{in}(x)}{dx^{2}}\right)+\frac{A_{55}^{in}}{r^{2}}\left(r\;\frac{dF_{3}^{in}(x)}{dx}+r^{2}F_{2}^{in}(x)-F_{1}^{in}(x)\right)+B_{11}^{in}\frac{d^{2}F_{2}^{in}(x)}{dx^{2}}\\ =\;I_{0}^{in}\;\omega^{2}\;\frac{\partial^{2}F_{1}^{in}(x)}{\partial t^{2}}+I_{1}^{in}\omega^{2}\;\frac{\partial^{2}F_{2}^{in}(x)}{\partial t^{2}} \end{array}$$

(24) $$\begin{array}{c} \frac{B_{11}^{in}}{r}\;\left(\;\frac{dF_{3}^{in}(x)}{dx}+r\;\frac{d^{2}F_{1}^{in}(x)}{dx^{2}}\right)-\frac{A_{55}^{in}}{r}\left(r\;\;\frac{dF_{3}^{in}(x)}{dx}-F_{1}^{in}(x)+rF_{2}^{in}(x)\right)+D_{11}^{in}\;\frac{d^{2}F_{2}^{in}(x)}{dx^{2}}\\ =I_{1}^{in}\;\omega^{2}\;\frac{\partial^{2}F_{1}^{in}(x)}{\partial t^{2}}+I_{2}^{in}\;\omega^{2}\;\frac{\partial^{2}F_{2}^{in}(x)}{\partial t^{2}} \end{array}$$

(25) $$\begin{array}{c} \frac{A_{55}^{in}}{r}\left(\frac{dF_{1}^{in}(x)}{dx}+r\frac{d^{2}F_{3}^{in}(x)}{dx^{2}}+r\;\frac{dF_{2}^{in}(x)}{dx}\right)-\frac{A_{11}^{in}}{r^{3}}\left(F_{3}^{in}(x)+r^{2}\frac{dF_{1}^{in}(x)}{dx}\right)+\frac{B_{11}^{in}}{r}\frac{dF_{2}^{in}(x)}{dx}\\ =\;I_{2}^{in}\;\omega^{2}\;\frac{\partial^{2}F_{3}^{in}(x)}{\partial t^{2}} \end{array}$$

(26) $$\begin{array}{c} \frac{A_{11}^{out}}{R}\left(\frac{dF_{3}^{out}(x)}{dx}+R\;\;\frac{d^{2}F_{1}^{out}(x)}{dx^{2}}\right)+\frac{A_{55}^{out}}{R^{2}}\left(R\;\frac{dF_{3}^{out}(x)}{dx}+R^{2}F_{2}^{out}(x)-F_{1}^{out}(x)\right)\\ +B_{11}^{out}\frac{d^{2}F_{2}^{out}(x)}{dx^{2}}\;=\;I_{0}^{out}\;\omega^{2}\;\frac{\partial^{2}F_{1}^{out}(x)}{\partial t^{2}}+I_{1}^{out}\omega^{2}\;\frac{\partial^{2}F_{2}^{out}(x)}{\partial t^{2}} \end{array}$$

(27) $$\begin{array}{c} \frac{B_{11}^{out}}{R}\;\left(\;\frac{dF_{3}^{out}(x)}{dx}+R\;\frac{d^{2}F_{1}^{out}(x)}{dx^{2}}\right)-\frac{A_{55}^{out}}{R}\left(R\;\;\frac{dF_{3}^{out}(x)}{dx}-F_{1}^{out}(x)+R\;F_{2}^{out}(x)\right)\\ +D_{11}^{out}\;\frac{d^{2}F_{2}^{out}(x)}{dx^{2}}\;=I_{1}^{out}\;\omega^{2}\;\frac{\partial^{2}F_{1}^{out}(x)}{\partial t^{2}}+I_{2}^{out}\;\omega^{2}\;\frac{\partial^{2}F_{2}^{out}(x)}{\partial t^{2}} \end{array}$$

(28) $$\begin{array}{c} \frac{A_{55}^{out}}{R}\left(\frac{dF_{1}^{out}(x)}{dx}+R\frac{d^{2}F_{3}^{out}(x)}{dx^{2}}+R\;\frac{dF_{2}^{out}(x)}{dx}\right)-\frac{A_{11}^{out}}{R^{3}}\left(F_{3}^{out}(x)+R^{2}\frac{dF_{1}^{out}(x)}{dx}\right)\\ +\frac{B_{11}^{out}}{R}\frac{dF_{2}^{out}(x)}{dx}=\;I_{2}^{out}\;\omega^{2}\;\frac{\partial^{2}F_{3}^{out}(x)}{\partial t^{2}} \end{array}$$

To solve aforementioned GEMs, the GDQ method is considered. This method is known for its high accuracy in estimating the NVFs of structures. The solution procedure begins by discretizing the problem domain into a series of grid points. The GDQ method represents the derivatives at each grid point as a linear combination of the function values at all other grid points in the domain. The corresponding weighting coefficients are determined using the following formula:

(29) $$P_{i}(x)\;=\;\frac{p(x)}{\left(x-x_{i})\;p^{\left(1\right)}(x\right)},\;i=1,\;2,\;\ldots ,\;N\;$$

Here,

(30) $$p\;(x)=\prod_{i=1}^{N}(x-x_{i}),\;p^{\left(1\right)}(x)=\prod_{i=1,\;i\neq j}^{N}\left(x_{i}-x_{j}\right)$$

With some mathematical work, the weighting coefficient for the first- and higher-order derivatives (*n*) can be obtained by utilizing the following equations:

(31) $$a_{ij}^{\left(1\right)}=\left\{\begin{array}{c} \;\frac{w^{\left(1\right)}(x_{i})}{\left(x_{i}-x_{j})\;w^{\left(1\right)}(x_{i}\right)},\;when\;\;i\neq j\\ -\sum_{k=1,\;j\neq i}^{N}a_{ik}^{\left(1\right)},\;\;\;\;\;when\;\;i=j \end{array}\right.,\;a_{ij}^{\left(n\right)}=\left\{\begin{array}{c} \;n\left(c_{ii}^{\left(n-1\right)}\;c_{ij}^{\left(1\right)}-\frac{c_{ij}^{\left(n-1\right)}}{x_{i}-x_{j}}\right),\;when\;\;i\neq j\\ -\sum_{k=1,\;j\neq k}^{N}c_{ik}^{\left(n\right)},\;\;\;\;\;\;\;when\;\;i=j \end{array}\right.\;$$

To discretize the domain of the CBR, the Chebyshev–Gauss–Lobatto method is considered. Therefore,

(32) $$\begin{array}{l} \Upsilon_{i}=\frac{1}{2}\left(1-\cos\left(\frac{i-1}{N-1}\pi \right)\right),\;\;\;i=1,\;2,\;\ldots ,\;N\\ x_{i}=\Upsilon_{i}\;\left(x_{f}-x_{0}\right)+x_{0},\;\;i=1,\;2,\;\ldots ,\;N \end{array}\;$$

Here, *x*_0_ and *x_f_* represent the coordinates of the system’s domain boundaries, marking the start and end points. Once the grid points are distributed across the domain and the corresponding weighting coefficients are calculated for each point, the effective stiffness matrix (*D_eff_*) and the mass matrix (*M*) are constructed. These matrices are assembled by combining the coefficients of the GEMs for each function, $F_{j}^{i}$(*x*), and are defined as follows:

(33) $$\left[D_{eff}\right]=\left[D_{I}\right]-\left[D_{IB}\right]\;{\left[D_{B}\right]}^{-1}\left[D_{BI}\right]\;$$

(34) $$\left[M_{eff}\right]=\left[M_{I}\right]$$

Here, *D_I_* is the stiffness matrix related to internal points, *D_IB_* is the internal stiffness matrix associated with BCs, and *D_B_* is the BC’s matrix stiffness. *M_I_* is defined as the mass matrix related to the internal points. Then, *ω* of the CBR can be determined by solving the following eigenvalue:

(35) $$\left|D_{eff}-\omega^{2}\;M_{eff}\right|=0\;$$

The NVFs of the CBR can be determined by using the following equation:

(36) $$NFs=\frac{2\pi }{\omega }$$

## 3. Nanocomposite Materials

**Figure 4:**
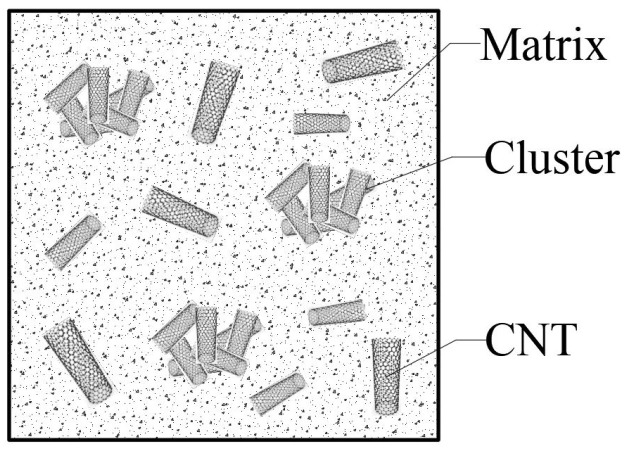
A schematic of agglomeration in clusters in a beam section.

The material considered for the curved beam is a composite reinforced with CNTs, which takes into account the agglomeration effect of these fibers. It is important to note that the distribution of CNT fibers within the matrix material is not consistently uniform. In specific regions of the beam, an aggregation of these CNTs occurs, resulting in the formation of clusters. To accurately model this phenomenon, the Shelby cluster model has been utilized. Figure 4 illustrates a schematic representation of this clustering within the representative volume element of the curved beam.

In this study, only the relationships proposed by Bui et al. [24] for calculating the effective elastic modulus and effective shear modulus (predicated on the homogenization of the material as an equivalent homogeneous material) have been utilized. The total volume of CNTs within the beam can be partitioned into two components, as follows:

(37) $$V=V_{c}^{t}+V_{m}^{t}$$

Here, *V_t_^c^* represents the volume of CNTs within a cluster, and *V_t_^m^* denotes the volume of CNTs in the matrix material, external to the clusters. The effective bulk and shear moduli of the cluster, as well as the effective bulk and shear moduli of the equivalent matrix material outside the clusters, are calculated as follows:

(38) $$K_{in}=K_{m}+\frac{V_{cnt}\eta \left(\delta_{r}-3K_{m}\alpha_{r}\right)}{3(\mu -V_{cnt}\eta +V_{cnt}\eta \alpha_{r})}$$

(39) $$K_{out}=K_{m}+\frac{V_{cnt}(1-\eta )\left(\delta_{r}-3K_{m}\alpha_{r}\right)}{3[1-\mu -V_{cnt}(1-\eta )+V_{cnt}(1-\eta )\alpha_{r}]}$$

(40) $$G_{in}=G_{m}+\frac{V_{cnt}\eta \left(\psi_{r}-2G_{m}\beta_{r}\right)}{3(\mu -V_{cnt}\eta +V_{cnt}\eta \beta_{r})}$$

(41) $$G_{out}=G_{m}+\frac{V_{cnt}(1-\eta )\left(\psi_{r}-2G_{m}\beta_{r}\right)}{2[1-\eta -V_{cnt}(1-\eta )+V_{cnt}(1-\eta )\beta_{r}]}$$

In these formulations, *V_cnt_* represents the volume fraction of nanotubes within the beam. The symbol *μ* represents the volume fraction of clusters to the total representative volume element, while *η* denotes the volume fraction of CNTs within the clusters to the total CNTs in the representative volume element. The subscripts *m* and *r* in the above relationships correspond to the matrix material and the reinforcing phase, respectively. The symbols *K_m_* and *G_m_* denote the bulk and shear moduli of the matrix material, respectively. The remaining parameters are defined as follows:

(42) $$\alpha_{r}=\frac{3(G_{m}+K_{m})+k_{r}-l_{r}}{3(G_{m}+k_{r})}$$

(43) $$\beta_{r}=\frac{1}{5}[\frac{\left(4G_{m}+2k_{r}+l_{r}\right)}{3(G_{m}+k_{r})}+\frac{4G_{m}}{G_{m}+p_{r}}+\frac{2G_{m}\left(G_{m}+3K_{m}\right)+2G_{m}\left(7G_{m}+3K_{m}\right)}{G_{m}\left(G_{m}+3K_{m}\right)+m_{r}\left(7G_{m}+3K_{m}\right)}]$$

(44) $$\delta_{r}=\frac{1}{3}[n_{r}+2l_{r}\frac{\left(2k_{r}+l_{r}\right)\left(2G_{m}+3K_{m}-l_{r}\right)}{G_{m}+k_{r}}]$$

(45) $$\psi_{r}=\frac{1}{5}[\frac{2}{3}(n_{r}-l_{r})+\frac{8G_{m}p_{r}}{G_{m}+p_{r}}+\frac{8G_{m}m_{r}\left(4G_{m}+3K_{m}\right)}{G_{m}\left(G_{m}+7m_{r}\right)+3K_{m}\left(G_{m}+m_{r}\right)}+\frac{2\left(2G_{m}+l_{r}\right)\left(k_{r}-l_{r}\right)}{3(G_{m}+k_{r})}]$$

The Hill elastic moduli for the reinforcing phase are defined as *k_r_* = 30 GPa, *m_r_* = 1 GPa, *n_r_* = 450 GPa, *p_r_* = 1 GPa, and *l_r_* = 10 GPa. The effective bulk and shear moduli of the composite material are determined using the following approach:

(46) $$K=K_{out}\left(\frac{\mu \left(\frac{K_{in}}{K_{out}}-1\right)}{\alpha \left(1-\mu \right)\left(\frac{K_{in}}{K_{out}}-1\right)+1}+1\right)$$

(47) $$G=G_{out}\left(\frac{\mu \left(\frac{G_{in}}{G_{out}}-1\right)}{\beta \left(1-\mu \right)\left(\frac{G_{in}}{G_{out}}-1\right)+1}+1\right)$$

where

(48) $$V_{out}=\frac{-2G_{out}+3K_{out}}{2G_{out}+6K_{out}}$$

(49) $$a=\frac{V_{out}+1}{3-3V_{out}}$$

(50) $$\beta =\frac{8-10V_{out}}{15-15V_{out}}$$

Finally, the effective elastic modulus, effective Poisson’s ratio, and equivalent density can be calculated using the following equations:

(51) $$E=\frac{9GK}{G+3K}$$

(52) $$\nu =\frac{-2G+3K}{2G+6K}$$

(53) $$\rho_{eff}=V_{cnt}\rho_{cnt}+V_{m}\rho_{m}$$

## 4. Validation

In this section, the accuracy of the formulated curved element is verified. The free vibration frequencies obtained from the analysis of a circular arc with clamped–clamped support conditions are compared with the corresponding results derived using the finite element method, thereby evaluating the accuracy of the proposed element. The mechanical properties of the homogeneous material used for this beam are as follows: *E* = 200 GPa, *ν* = 0.34, and *ρ* = 7850 kg/m³. The central angle of the beam is π/4, and its cross-section is rectangular. The geometric characteristics of the beam include R = 7.5 m and h = 0.1 m, with the width of the cross-section is equal to its height. To determine the first six NVFs, the beam was discretized using 15 and 30 elements. Table 1 presents the NVFs obtained using the proposed method in this study, alongside the results reported by Mottaghi et al. [16]. As observed, the element employed in this research is capable of accurately computing the NVFs of the curved beam with high precision.

## 5. Parametric Studies

In this section, the effect of CNT incorporation on the strength of the composite material has been investigated. For this purpose, the base material is assumed to be conventional concrete with a density of *ρ* = 2777 kg/m^3^ and a characteristic compressive strength of 25 MPa. Considering a Poisson’s ratio of 0.2 for the concrete, the elastic modulus, shear modulus, and bulk modulus of the base material are calculated as *E* = 23,500 MPa, *G* = 9791.67 MPa, and *K* = 13,055.56 MPa, respectively [16].

**Figure 5:**
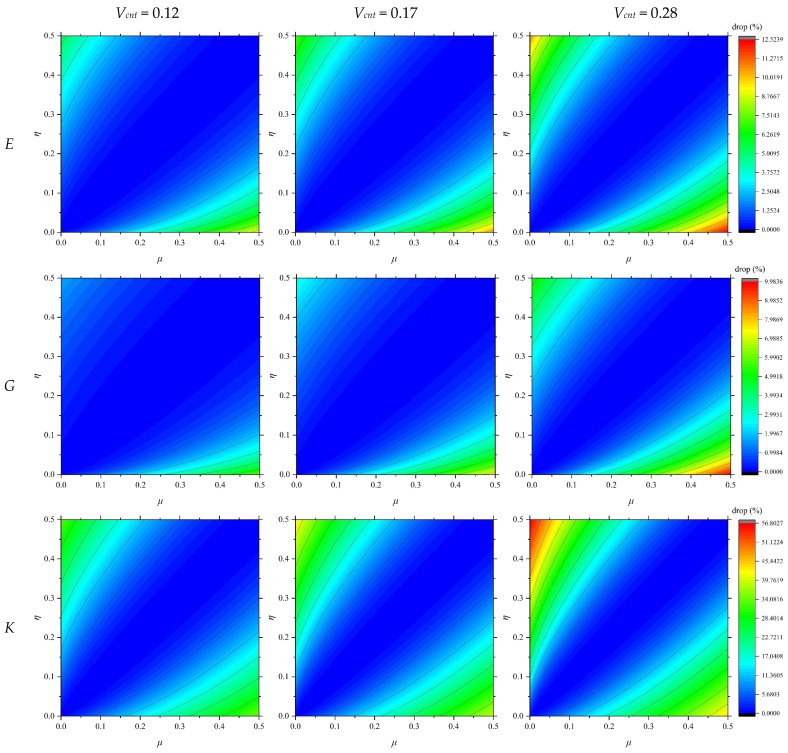
Reduction in the *E*, *G*, and *K* of the composite material with variation *V_cnt_*.

With the availability of these moduli, the elastic modulus, shear modulus, and bulk modulus of the CNT-reinforced composite material, accounting for the agglomeration effects, were calculated using the equations provided in Section 3. Figure 5 illustrates the impact of agglomeration factors on the reduction in the elastic, shear, and bulk moduli of the composite material for CNT volume fractions of 0.12, 0.17, and 0.28.

Based on the analysis of the data presented in Figure 5, the following conclusions were drawn:

- When the coefficients *μ* and *η* are not balanced relative to each other, the composite material exhibits significant reductions in the elastic, shear, and bulk moduli.
- As the ratio *μ*/*η* deviates further from unity, the extent of modulus reduction increases noticeably.
- At higher CNT volume fractions, the adverse effects of CNT shrinkage become more pronounced.

These findings highlight the critical importance of precisely controlling the coefficients and *η*, as well as the CNT content, in the design of composite materials to achieve optimal mechanical properties and mitigate potential negative effects.

**Figure 6:**
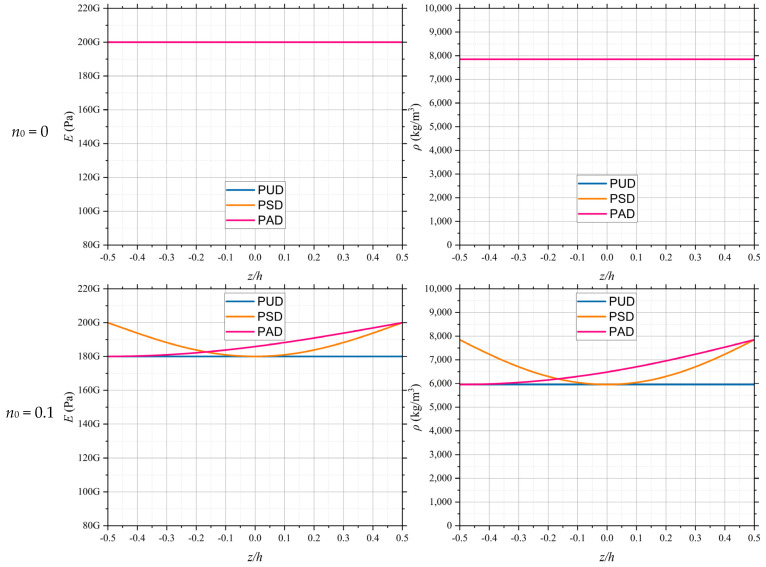

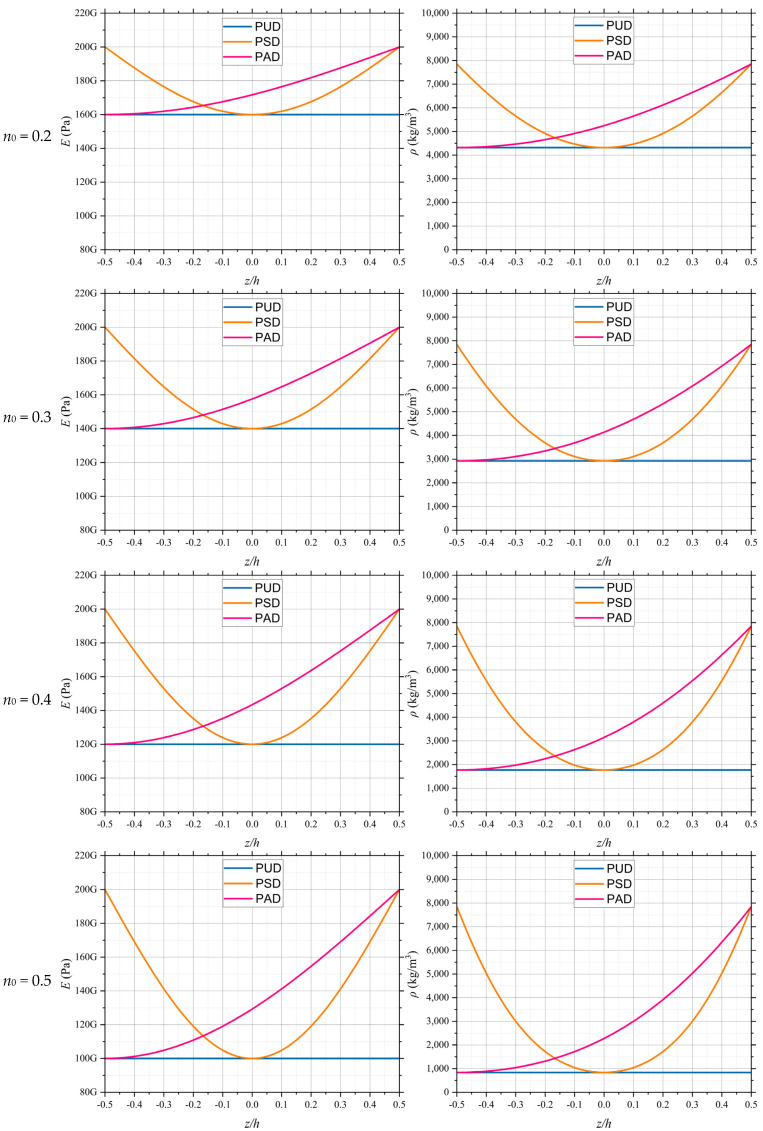
Equivalent *E* and *ρ* functions of the porous material for various initial porosity values.

In another example, the effect of initial porosity on the mechanical properties of the material was investigated under three conditions: uniform, symmetric, and asymmetric distributions. For this material, the following parameters were assumed: *E* = 200 GPa, *ν* = 0.34, *ρ* = 7850 kg/m^3^, and *κ* = 12.143 W/mK. Additionally, the thermal conductivity of air was considered to be 0.025 W/mK. Figure 6 illustrates the equivalent elastic modulus and density functions of the porous material for various initial porosity values. As observed in the figure, the elastic modulus and density remain constant for the uniform distribution case. In the symmetric distribution, the elastic modulus and density form a parabolic function, where the depth of the parabola increases with the rise in initial porosity. The values of these functions are symmetric at about *z* = 0, and the top sheet exhibits behavior similar to that of a homogeneous material. In the asymmetric distribution, based on the proposed modeling, the porosity reaches its maximum value at *z* = −0.5*h* and decreases to zero at *z* = 0.5*h*. Consequently, the values of the functions are at their minimum in the bottom sheet of the section and resemble those of a homogeneous material in the top sheet.

**Figure 7:**
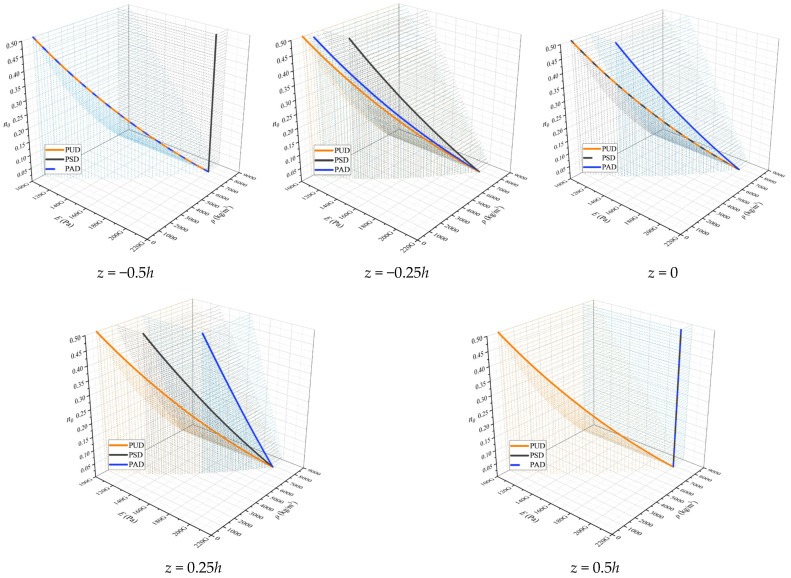
Values of *E* and *ρ* for a material for various *z*.

To provide a more precise analysis of the effect of porosity distribution, Figure 7 illustrates the values of elastic modulus and density for a material with mechanical properties similar to those in the previous example, across various initial porosity levels. This figure essentially emphasizes the findings of the previous example. Generally, under symmetric distribution, the maximum values of elastic modulus and density are observed in the top and bottom sheet of the section, where porosity is zero, while the minimum values occur in the mid-sheet of the section, similar to the uniform distribution case. Conversely, under asymmetric distribution, the porosity in the bottom sheet of the section remains similar to that of the uniform distribution and gradually decreases, such that the porosity in the top sheet of the section becomes zero. Consequently, the elastic modulus and density reach their minimum values in the bottom sheet and their maximum values in the top sheet of the section.

## 6. Numerical Investigation

**Figure 8:**
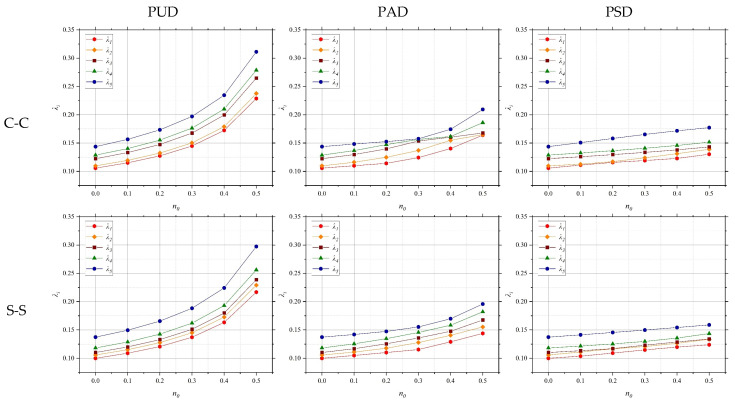
NVFs of QCNCR for different porosity distribution patterns with C-C and S-S BCs.

The influence of porosity distribution patterns on the NVFs of the system under various BCs is investigated. Figure 8 illustrates the dimensionless frequencies (DFs) of QCNCR for different porosity distribution patterns. The geometric parameters of the arcs are *R* = 1 m, *h*= 0.1 m, and *α* = π/4. Also, the mechanical parameters of the rings are *E* = 200 GPa, *ν* = 0.34, *ρ* = 7850 kg/m^3^, and *κ* = 12.143 W/mK. The thermal conductivity of air is *κ*_air_= 0.025 W/mK. As shown, the DFs increase with the porosity coefficient. This trend can be attributed to the reduction in mass, which dominates over the accompanying decrease in stiffness, leading to higher vibrational frequencies. Furthermore, the relationship between DFs and the porosity coefficient is pattern-dependent. For symmetric and asymmetric porosity distributions, the relationship exhibits nonlinear behavior due to the non-uniform mass and stiffness variations influencing the dynamic response. In contrast, for a uniform porosity distribution, the relationship remains linear, as both mass and stiffness are reduced in a proportionally consistent manner.

**Figure 9:**
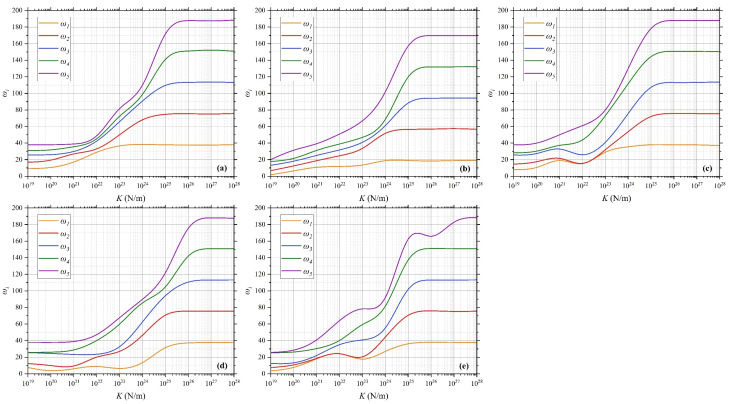
NVFs for various spring stiffness values under different BCs: (**a**) C-C/C-C, (**b**) C-F/C-F, (**c**) C-S/C-S, (**d**) S-S/S-S, and (**e**) SD-SD/SD-SD.

The QCNCR system consists of two curved beams interconnected by a layer of elastic springs. The stiffness of these springs plays a crucial role in determining the NVFs of the system. Figure 9 illustrates the NVFs for various spring stiffness values under different BCs. The geometric parameters of the arcs are *R*_1_ = 1 m, *R*_2_ = 0.6667 m, *h* = 0.1 m, and *α* = π/4. The material properties of matrix and CNTs are shown in Table 2.

As observed, the NVFs increase with increasing K, exhibiting a nonlinear trend. For relatively low stiffness values, the NVFs vary gradually, as the elastic springs provide limited resistance to deformation, allowing greater flexibility in the system’s response. However, as the stiffness increases (K > 10^25^ N/m), the NVFs asymptotically reach constant values, indicating a transition toward a nearly rigid connection. In this regime, the springs effectively act as rigid constraints, significantly restricting relative motion between the beams and resulting in vibrational characteristics similar to those of a monolithic structure.

## 7. Conclusions

In this study, the free vibration behavior of CNT-reinforced composite ring structures was investigated, incorporating FSDT and Hamilton’s principle for modeling. The analysis considered the effects of CNT agglomeration, porosity distribution, and spring stiffness on the system’s dynamic response. The findings contribute to the optimization of composite ring structures for various engineering applications, including aerospace, mechanical, and vibration-sensitive systems. Key conclusions drawn from this investigation are as follows:

- CNT agglomeration significantly reduces the composite’s elastic, shear, and bulk moduli, leading to lower NVFs.
- Porosity distribution patterns strongly influence the NVFs, with asymmetric distributions exhibiting nonlinear trends due to mass and stiffness variations.
- Increasing the stiffness of elastic springs results in higher NVFs, eventually stabilizing at rigid connection levels.
- The balance between agglomeration parameters (μ and η) is crucial for maintaining optimal mechanical performance.
- Coupled and nested ring structures exhibit enhanced vibrational characteristics, making them suitable for applications in precision devices and structural damping mechanisms.

## Figures and Tables

**Table 1:** The NVFs of beams for C-C BCs.

Number of Modes	15 Nodes		30 Nodes	
	GDQM	FEM [16]	GDQM	FEM [16]
1	40.2788	40.5094	40.2788	40.0262
2	67.0537	67.9384	67.0537	66.8919
3	106.269	106.748	106.269	106.182
4	131.019	135.122	131.020	131.034
5	197.798	206.665	197.801	198.161
6	271.957	288.052	270.888	271.810

**Table 2:** Mechanical properties of SWCNTs (10,10) and PMMA (reprinted from ref. [25]).

Item	Value	Unite
SWCNT (10,10)		
*E^matrix^*	2.5	GPa
*ν^matrix^*	0.34	---
*ρ^matrix^*	1190	Kg/m^3^
*κ^matrix^*	0.168	W/mK
*α^matrix^* (at 300 K)	45 × 10^−6^	---
PMMA		
*E_11_^cnt^*	600	GPa
*E_22_^cnt^*	10	GPa
*G^cnt^*	17.2	GPa
*ν^cnt^*	0.19	---
*ρ^cnt^*	1400	Kg/m^3^
*κ^cnt^*	2000	W/mK
*α^cnt^* (at 300 K)	3.4584 × 10^−6^	---

## Data Availability

Data is contained within the article.

## References

[1] Abedinilaksar M., Yang J. (2023). Free in-plane vibration of thin-walled rings with elastic supports. J. Mech. Sci. Technol..

[2] Wu X., Parker R.G. (2006). Vibration of rings on a general elastic foundation. J. Sound Vib..

[3] Xu G.-H., Huang H.-W., Zhang Y.-Q. (2017). Vibration of Elastic Functionally Graded Thick Rings. Shock. Vib..

[4] Zhipeng W., Wei L., Yunbo Y., Zhijun S., Yibin G., Donghua W. (2018). Free Vibration Analysis of Rings via Wave Approach. Shock. Vib..

[5] Zhang K., Miao Z., Xia H., Yang X., Tian F., Zhao Y. (2025). Effects of Repair Interface Structure on Mechanical Properties of Scarf Repair for Composite Laminate Plates. Polymers.

[6] Roy S., Thakur S.N., Ray C. (2021). Free vibration analysis of laminated composite hybrid and GFRP shells based on higher order zigzag theory with experimental validation. Eur. J. Mech.-A Solids.

[7] Garg A., Shukla N.K., Belarbi M.-O., Barnawi A.B., Raman R., Sharma A., Li L. (2024). Free vibration analysis of bio-inspired helicoidal laminated composite square and annular plates having circular openings using isogeometric analysis. Structures.

[8] Karamanli A., Vo T.P., Belarbi M.-O., Lee S. (2025). On the bending, buckling and free vibration analysis of bio-inspired helicoidal laminated composite shear and normal deformable beams. Compos. Struct..

[9] Ghandehari M.A., Masoodi A.R., Hosseininia S.E.S. (2024). Multiscale dynamic behavior of imperfect hybrid matrix/fiber nanocomposite nested conical shells with elastic interlayer. Thin-Walled Struct..

[10] Jiang D., Zhao Y., Guo R., Chen M. (2024). Analysis of dynamic modeling and power flow of ABH laminated composite thin beam with elastic boundaries. Thin-Walled Struct..

[11] Deng E.-F., Yang Y.-M., Tian Y., Zhang Z., Wang Y.-B., Wei J.-Z., Dong S.-L. (2024). Flexural behavior of laminated H-beams in modular constructions: Numerical and analytical studies. Structures.

[12] Zhang Y., Shi D., He D., Shao D. (2021). Free Vibration Analysis of Laminated Composite Double-Plate Structure System with Elastic Constraints Based on Improved Fourier Series Method. Shock. Vib..

[13] Sahib M.M., Kovács G. (2025). Using Artificial Neural Networks to Predict the Bending Behavior of Composite Sandwich Structures. Polymers.

[14] Alkhoury P., Aït-Ahmed M., Soubra A.-H., Rey V. (2022). Vibration reduction of monopile-supported offshore wind turbines based on finite element structural analysis and active control. Ocean. Eng..

[15] Avey M., Fantuzzi N., Sofiyev A.H., Kuruoglu N. (2021). Nonlinear vibration of multilayer shell-type structural elements with double curvature consisting of CNT patterned layers within different theories. Compos. Struct..

[16] Mottaghi T.H., Masoodi A.R., Gandomi A.H. (2024). Multiscale analysis of carbon nanotube-reinforced curved beams: A finite element approach coupled with multilayer perceptron neural network. Results Eng..

[17] Liu J., Li X., Liu J., Xu Y., Pan G. (2025). Vibration analysis of a coupled propulsion shaft-shell system based on the numerical and semi-analytical methods. J. Sound Vib..

[18] Thang P.T., Kim C., Jang H., Kim T., Kim J. (2025). Free vibration characteristics of honeycomb sandwich cylindrical shells reinforced with graphene nanoplatelets/polymer coatings. Aerosp. Sci. Technol..

[19] Charoensuk K., Sethaput T. (2023). The Vibration Analysis Based on Experimental and Finite Element Modeling for Investigating the Effect of a Multi-Notch Location of a Steel Plate. Appl. Sci..

[20] Guo W., Feng Q. (2019). Free Vibration Analysis of Arbitrary-Shaped Plates Based on the Improved Rayleigh–Ritz Method. Adv. Civ. Eng..

[21] Hao Y., Sun L., Zhang W., Li H. (2025). Active traveling wave vibration control of elastic supported conical shells with smart micro fiber composites based on the differential quadrature method. Appl. Math. Mech..

[22] Hao Y.X., Li H., Zhang W., Ge X.S., Yang S.W., Cao Y.T. (2022). Active vibration control of smart porous conical shell with elastic boundary under impact loadings using GDQM and IQM. Thin-Walled Struct..

[23] Ghandehari M.A., Masoodi A.R., Hosseininia E.S. (2025). Temperature-dependency and boundary condition impacts on the multiscale vibrational behavior of laminated nested dual conical shell structure semi-AUV applications. Appl. Ocean. Res..

[24] Bui T.T.H., Tran T.T., Nguyen D.K. (2022). Geometrically nonlinear analysis of sandwich composite beams reinforced by agglomeration carbon nanotubes. Vietnam. J. Mech..

[25] Ghandehari M.A., Masoodi A.R. (2024). Inherent resonance of carbon and graphene-based nanocomposite coupled single-span arch beams. Compos. Part C Open Access.

